# Children’s Perceived and Actual Physical Activity Levels within the Elementary School Setting

**DOI:** 10.3390/ijerph18073485

**Published:** 2021-03-27

**Authors:** Kristy Howells, Tara Coppinger

**Affiliations:** 1 Department of Sport, Exercise and Rehabilitation Sciences, School of Psychology and Life Sciences, Faculty of Science, Engineering and Social Sciences, Canterbury Christ Church University, Canterbury CT1 1QU, UK; 2 Department of Sport, Leisure & Childhood Studies, Faculty of Business & Humanities, Munster Technological University, T12 P928 Cork, Ireland; Tara.Coppinger@cit.ie

**Keywords:** physical activity, children, perceived activity levels, elementary school

## Abstract

To date, little research has longitudinally examined young children’s physical activity (PA) during school hours, nor questioned children’s perceptions of their own PA behaviours. This study investigated 20 children’s actual physical activity levels (APA) and their perceived physical activity levels (PPA) (10 infants, mean age 6.6 years; 10 juniors, mean age 9.5 years). APA was evaluated using accelerometers across 36 whole school days (371 min per day); 18 days included Physical Education (PE) lessons and 18 did not. A repeated-measures three-factor ANOVA analysed: type of day; age phase; parts of the day and sex. PPA was collected by an interactive handset and an adapted version of the PA Questionnaire for Children (PAQ-C). Participants undertook 10 more minutes of moderate to vigorous PA (MVPA) on PE days (53 ± 19 min) compared to non-PE days (43 ± 15 min) (F = 92.32, *p* < 0.05) and only junior boys reached daily MVPA recommendations (60 ± 13 min) on PE days. Juniors over-estimated, and infants under-estimated, their APA levels. Educators need more support to teach and embed different PA intensities into the school day to enable children to better understand the health benefits associated with varying the intensity of their PA during school hours.

## 1. Introduction

The purpose of this study is to examine children’s actual physical activity (APA) levels and their perceived physical activity (PPA) within an English elementary school setting, during the 9 am–3.10 pm structured school day. This study investigated whether there were any differences between children’s APA versus PPA, with the purpose of potentially addressing ways to reduce any levels of mismatch. Comparisons were also made between Physical Education (PE) days and non-PE days, as PE tends to only take place twice a week in English elementary schools. Most recent research has been limited to a short time frame, children aged 9 years and older and few have focused on physical activity during school hours. 

From an educational perspective, it is important for teachers to know what level of understanding children have about their own physical activity and how their bodies are responding to its different intensities. By potentially increasing an understanding of physical activity levels, this may help children move towards more autonomy and self-governance of their own daily physical activity levels, whilst also supporting them to meet the recommended daily levels [1,2].

International research has indicated observing and understanding one’s own physical activity correctly is universally difficult for children [3]. This difficulty between PPA and APA has been identified in a variety of ways, most recently when young children are learning aquatic skills [4], as well as inconsistencies in their accuracy to estimate their ability to perform physical tasks [5].

Parents have also been found to wrongly perceive the physical activity levels of their children, with one study in Britain finding most parents of inactive children to incorrectly report their children as sufficiently active [6]. This mismatch between perceived and APA levels, physical tasks and motor competencies identifies how difficult recognising and understanding movement can be, particularly for young children. Yet, as children age (10 years+), their ability to accurately report motor competency and physical activity level tends to increase [7,8]. Supporting younger children to further their understanding of their APA might therefore be an important consideration; given that it is during this life stage that lifelong physical activity behaviours are formed [9].

Although the UK Government made a long term commitment to reduce physical inactivity as part of the legacy from the London 2012 Games [10], a report by ukactive [11] in 2018 stated that only 1 in 4 boys and 1 in 5 girls reached the recommended 60 min of moderate to vigorous physical activity (MVPA) per day [1,2]. The Olympic legacy has, ultimately, failed and the nation’s children have aptly been labelled as the ‘least active generation ever’ [11]. It could be argued that a lack of understanding between APA and PPA could be the cause. This could be being exacerbated further due to the lack of support teachers receive when trying to fully implement physical activity ‘for sustained periods of time’ as part of the National Curriculum for PE in England (p. 198) [12]. No specific curriculum guidance is currently provided to teachers, parents or children on to how sustain physical activity [12] nor how to lead active lives. 

Physical health and fitness is featured within the health education element of the new (2020/21) Relationships Education, Relationships and Sex Education and Health Education curriculum in England [13]. Elementary age children are due to learn about ‘the physical benefits of active lifestyles’ (p. 34) [13], how to achieve regular vigorous exercise and the risks of inactivity. Although the UK’s Chief Medical Officers [1] identified PE as ‘likely to be a key role in the development of movement skills and… is important for the development of children’s skills and confidence to be physically active’ (p.27) [1], it is quite possible that elementary teachers and early years practitioners do not fully understand their importance. This lack of specific knowledge may further impact children’s physical activity levels during the ongoing global pandemic. Limited training is provided on physical activity and motor competence in UK initial teacher training courses [14] and many may lack the confidence to embed movement integration [15] away from scheduled PE time [16]. In order to ascertain whether children do, in fact, incorrectly report their physical activity levels, this study aims to analyse younger (6–7 years old (infants)) and older (9–10 years old (juniors)) children’s perceived and APA levels within the elementary school setting. Particular attention will be paid to compare any differences according to age phase, sex and structure of the school day. 

## 2. Materials and Methods 

### 2.1. Participants

A total of 20 children (5 boys and 5 girls (aged between 6–7 years old, referred to as infants, mean age = 6 years 6 months); 5 boys and 5 girls (aged between 9–10 years old, referred to as juniors, mean age = 9 years 5 months)) from one rural village faith (Church of England) elementary school in the South East of England participated in this study. These two different age ranges were selected to capture an insight into the different English national curriculum phases (infants and juniors) that the elementary school is split into [12]. The children were from the same socio-economic backgrounds (middle class), as derived from the National Census [17], and the school had achieved a ‘Healthy School Status Mark,’ which aims to seek to achieve healthy lifestyles for the entire school population [18]. 

The longitudinal case study design allowed for a 7 month analysis (January–July) and for 36 days of data to be collected from each child (18 days were PE days and 18 days were non-PE days, i.e., days that did not include a PE lesson). The purpose of comparison of the two types of days (PE day versus no PE day) was to consider the potential impact that PE can have on children’s overall physical activity levels during the school day. Within the English context, elementary schools often only have 2 days in the school week where children partake in PE. A longitudinal case study design was chosen as Pearce et al. [19] highlighted that physical activity is variable and it is difficult to assess and measure as it tends to “change with days, weeks, seasons” (p. 169). The majority of previous research on children’s physical activity has been over a limited timeframe [20]; only allowing for a snapshot of what is occurring. 

Most class teachers within elementary schools in England are known as ‘generalist teachers’, who teach all day with the same class. Within most initial teacher education programs, trainee teachers specialise within one subject. The class teachers involved in this research had over 25 years teaching experience between them and were both Primary PE [21] specialists, who had taken extra training within their initial teacher training programs. These teachers were chosen to overcome any limitation caused by a lack of confidence in delivering PE and/or embedding physical activity into the school day [15]. A pilot study, which included 3 days of PE and 3 days of no PE during September-December, was administered to allow each child to become familiar with the physical activity measurement tool (accelerometers). All children in both age groups completed the pilot study and no data collection days were missed. Ethical approval was granted from Canterbury Christ Church University Research Ethics Committee (08/SAS/009) and all the participants details were anonymised prior to analysis. All parents gave their informed, written consent for their children to participate and the children gave their informed assent to take part, alongside the headteacher allowing for this study to be undertaken within their school setting. 

### 2.2. Procedure 

In order to prevent selection bias [22] and ensure an equal representation of each sex, consenting participants’ names were randomly drawn. The children were familiar with this transparent selection process as the teachers used it for answering questions within lessons daily. It is acknowledged that the sample selected was not fully random or stratified [23] and therefore only reflects the population of the two classes of the school, rather than the population on a whole [24]. No changes were made to the normal school day schedule to facilitate the current research and as the PA recordings occurred throughout the school day, children that were not participating still undertook the same activities as those of the participants. 

Due to young children’s cognitive-ability and recall limitations, direct observation or objective measures such as accelerometers should be used to evaluate physical activity levels in this age group [25]. Accelerometers are a valid and reliable tool for measuring the physical activity levels of children [26,27]. ActiGraph accelerometers (model 7161, Pensacola, FL, USA) were worn on an elastic belt around the waist [28] and measured the APA of participants, with the use of the ActiSoft software 3.2 system [29] for analysis. This type of uniaxial accelerometer assesses accelerations ranging from 0.05 to 2.0 G and has a band limited frequency response from 0.25 to 2.5 Hz [30]. The waist is the most preferred site as it accurately records locomotor movements and participants find it less obtrusive for carrying out routine daily activities [31,32]. Due to the longitudinal nature of this study, comfort and compatibility of the accelerometer were important considerations. The pilot phase allowed for repeated positioning and adjustment alterations of the waist belt to allow for habitual wearing of the accelerometer, as recommended by multiple researchers [33,34]. The children wore the accelerometers for 371 min a day, for 36 days, within the main longitudinal phase of the data collection (January–July). There were no wet recess playtimes (where children had to remain indoors in their classroom), no cancelled PE lessons and no missed data collection days. A 60 s epoch recording time and the cut points were used [30] as these have both been previously recommended when assessing the physical activity levels of children [33,34,35]. Additionally, the low extension frequency filter was turned on to ensure all of the children’s sporadic movements were captured. 

An adapted version of the Physical Activity Questionnaire for Children (PAQ-C) [36] was used to record the children’s PPA. Qwizdom technology [37], an interactive learning system that uses two-way infra-red communications to provide instant recording, was used to collect the responses. The children were already familiar with the handsets as they were often used as an interactive tool within their classroom environment. The questions were posed on a large screen via a laptop for the whole group to be able to see and they were also discussed one by one with the infant-aged children to assist with understanding. The handset was durable and instantly recorded but did not display the child’s answer [38] to reduce the effects of peer influence in terms of responses [39]. Interactive handsets encourage students to think about what they are doing [40] and can increase children’s attentiveness [41], important considerations for accurate questionnaire completion. 

### 2.3. Data Analysis

APA data were analysed for the overall school day (total = 371 min) and for each part of the school day (recess, lunch time, PE lessons and other curriculum time). The total minutes per part of the school day varied according to PE versus non-PE days (Table 1). Recess occurred in both the morning and afternoon for infants, but only in the morning for juniors. Recess and lunch times are unstructured times for schoolchildren, where they are free to undertake spontaneous free play and often provided with both small and large equipment (skipping ropes, balls). The case study school had access to two playgrounds, which had netball posts, basketball hoops, hopscotch grids and a balance trim trail (similar to an obstacle course), in which the children could practice developing their balance, control and coordination skills. The children also had access to a large playing field, which had football (soccer) goals on it. There was also large play adventure equipment on the field that had areas for climbing, swinging, sliding, as well as sand pits, and a ‘noughts and crosses’ (tic, tac, toe) game. The 6–7 year olds (infants) had a ‘free flow’ environment during their curriculum time and were allowed to engage in their learning either in the indoors or outdoors and could move freely from one area to another. The curriculum time for the younger children focused on an enquiry and exploratory based approach to learning that was recommended to support their processing skills and holistic learning [41]. The older children’s (9–10 year olds) curriculum time was more structured, whereby a more formal style of lessons was delivered with learning occurring mainly within the classroom indoor environment and with the children sitting in set seats, according to the subject being taught. PE lessons for both ages were structured lessons.

Data were analysed for intensity and presented for MVPA (≥3 METs) and light physical activity (LPA) (≥2 METs and <3 METs) across each part of the school day, in order to make comparisons with the questionnaire and children’s PPA. A repeated-measures three-factor analysis of variance (ANOVA) was used to analyse the effects of factors including the following: type of day (PE days/non-PE days), sex (boys/girls) year group (infants/juniors) and parts of the day (curriculum time/morning recess/lunch time/afternoon recess). For the PE lesson part of the day, a two-factor univariate ANOVA (year group and sex) was completed, as the type of day could not be compared due to there being no equivalent time within non-PE days. Statistical significance was set at <0.05 ± one standard deviation and statistical analysis was completed using SPSS 22.0 (IBM Corp, Armok, NY, USA). The data were converted from minutes to percentage number of minutes and was inputted into excel before analysis in SPSS 22.0 to enable direct comparison between infants and juniors, as their school days differed.

The adapted questionnaire examined the previous 7 days and was used to measure ‘general MVPA levels during the school year’ (p. 3) [36]. The importance of exploring the physical activity of the whole week for children has been regarded as valuable in terms of the information gained [42]. A Likert scale was used to collect the data, but with no middle option of neither like nor dislike, as this middle option was not used in the original PAQ-C questionnaire [36] and encouraged children to have an opinion. The number of responses for each answer were recorded and percentages calculated. Although not all questions from the questionnaire were needed to capture the school day, all the questions were asked, as by asking questions about all aspects of children’s physical activities, this helps to utilise their memory cues [36]. However, at the time of analysis, the physical activity in different settings questions/answers were therefore omitted. The questions focused on the children’s overall impression of how physically active they felt that they were (their PPA) and the data were collected through the following subthemes: (a) travelling to school; (b) recess time; (c) activities within PE; (d) activities completed within the last seven days; (e) activities they undertook during the previous weekend and (f) how physically active they would describe themselves. Therefore, the focus for the results within this paper will consider only subthemes b, c and f. 

## 3. Results

### 3.1. Actual Physical Activity (APA)

#### 3.1.1. Average MVPA Levels

**Figure 1:**
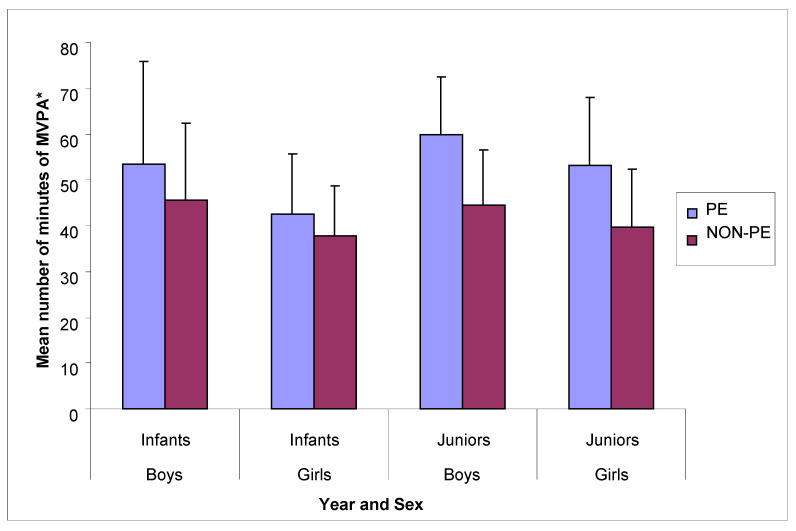
Children’s overall mean number of minutes ± SD of MVPA during PE and non-PE by year group and sex. * MVPA = moderate to vigorous physical activity levels. PE = Physical Education.

The data showed PE lessons to significantly contribute to children’s overall MVPA, with children completing, on average, 10 more minutes of MVPA on PE days (53 ± 19 min) compared to non-PE days (43 ± 15 min) (F(1, 16) = 92.32, *p* < 0.05). There was a significant main effect for sex, with boys (50 ± 17 min) completing 5 more minutes of MVPA on average (F(1, 16) = 9.04, *p* < 0.05) than girls (45 ± 13 min). There was also a significant interaction (F(1, 16) = 4.47, *p* < 0.05) with boys and juniors, who completed 11 min (55 ± 19 min) and 13 min (53 ± 15 min) more MVPA, respectively, on PE days when compared to non-PE days (44 ± 15 min; 40 ± 13 min). When the data were compared according to year group and sex, junior boys reached, on average, the recommended daily target of 60 min of MVPA [38] (60 ± 13 min) during school hours. However, this only occurred on PE days (Figure 1 and Table 1). 

#### 3.1.2. MVPA within Lunch Time and Recess

The different physical activity intensity levels were measured within recess and lunch times (Table 2). There were significant differences found for the type of day, with children, on average, completing 1 min more MVPA on PE days during recess compared to non-PE days (F(1, 16) = 18.96, *p* < 0.05). There was also a significant effect for sex with boys completing 1 min more MVPA during recess than girls (F(1, 16) = 22.02, *p* < 0.05) and, on average, on PE days infants completed 2 min more MVPA compared to juniors (F(1, 16) = 14.6, *p* < 0.05). During lunch times, juniors undertook, on average, 4 more minutes of MVPA than infants (F(1, 16) = 4.89, *p* < 0.05) and boys completed 2 min more MVPA than girls (F(1, 16) = 24.34, *p* < 0.05). For afternoon recess, girls were found to undertake 1 min more MVPA on non-PE days compared to PE days (F(1, 8) = 12.57, *p* < 0.05).

#### 3.1.3. LPA within Lunch Time and Recess

On average, infants (F(1, 16) =6.16, *p* < 0.05) undertook 2 min more (9 ± 3 min) of LPA compared to juniors (7 ± 2 min) during morning recess. Girls undertook 1 min more LPA compared to boys (F(1, 16) = 7.02, *p* < 0.05), whilst infants significantly completed 2 min more LPA on non-PE days versus PE days (F(1, 16) =14.81, *p* < 0.05). During lunch times girls undertook most of their activity in the LPA intensity on non-PE days (F(1, 16) =6.05, *p* < 0.05) and undertook 1 min more LPA on non-PE days (22 ± 4 min) compared to PE days (21 ± 3 min). Infant girls (F(1, 16) = 4.96, *p* < 0.05) undertook 2 min more LPA on non-PE days (23 ± 3 min) when compared to PE days (21 ± 2 min), whilst junior girls undertook 1 min more LPA on PE days (21 ± 4 min) when compared to non-PE days (20 ± 4 min). There were no significant differences in the amount of LPA during afternoon recess; similar levels were found for both sex and type of day.

### 3.2. Perceived Physical Activity (PPA)

#### 3.2.1. How Active Do You Think You Are during Recess?

**Figure 2:**
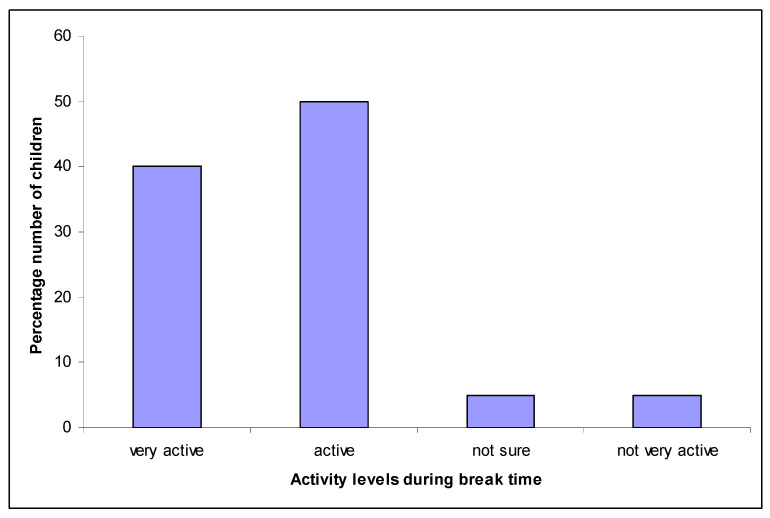
Children’s perception of how active they are during recess (break time).

The most popular response by children was that they described themselves as being ‘active’ during recess (50% *n* = 10), (Figure 2), with ‘very active’ being the second most reported response (40% *n* = 8). Only 5% (*n* = 1) of children described themselves as ‘not very active’, whilst 5% (*n* = 1) were ‘not sure’ how active they were during recess (both morning and afternoon, for infants). The accelerometer data indicated that children over-estimated their intensity levels during recess, as, on average, only 50% of recess time was at the MVPA level.

#### 3.2.2. Which Activities Do You Participate in during Lunch Time?

**Figure 3:**
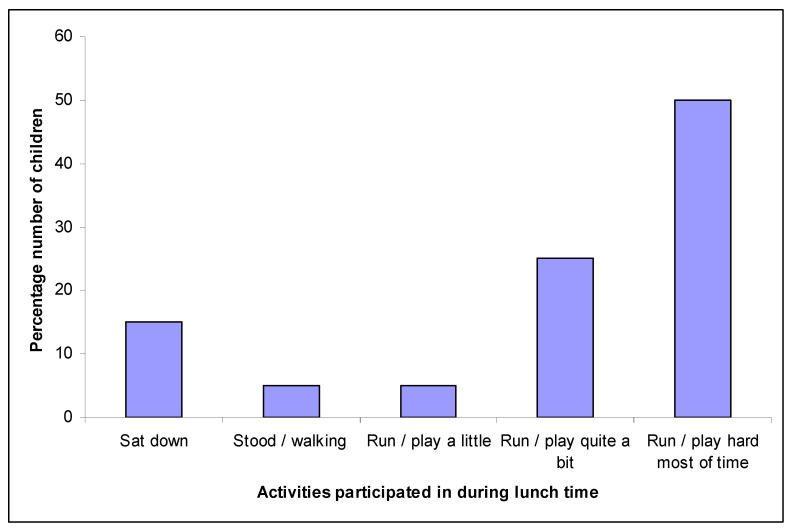
Most popular reported activities reported by children during their lunch time.

When asked what activities they undertook at lunch time (other than eating their lunch), 50% (*n* = 10) reported that they ‘ran and played hard for most of the time’, whilst 25% (*n* = 5) reported that they did this for ‘quite a bit of time’ (Figure 3). A total of 15% (*n* = 3) of children reported their lunch time activities to be more inactive and included more stationary behaviour such as sitting down, talking, reading or doing some school work. The lunch time accelerometer data again showed an over-estimation by children. The APA indicated that only 25% of lunch time was spent at a MVPA level.

#### 3.2.3. How Frequently Are You Very Active within PE Lessons?

Children were asked about their physical activity levels during PE and how often they thought that they were very active (playing hard, running hard, and jumping). The most popular response was that children felt that they were ‘quite often’ (60%; *n* = 12) very active in PE lessons. Over 1/3 (35%; *n* = 7) described themselves as ‘very active’ within PE lessons. Girls described themselves as ‘quite often active’, whilst boys described themselves as both ‘very active’ and ‘quite often very active’. There were no significant differences in APA intensity during PE lessons. 

## 4. Discussion

This paper aimed to analyse younger (6–7 year olds) and older (9–10 year olds) children’s APA and PPA levels within the elementary school setting. When children’s APA and PPA were compared, there were some similarities in their knowledge and understanding, but the data indicate many discrepancies between APA and PPA. Ultimately, older children (juniors) overestimated and younger children (infants) under-estimated their APA levels. These data reflects earlier work [43], which identified age differences when children estimate their PA levels. Measuring physical activity of very young children has previously been identified as challenging [44] and Haskell [45] earlier recommended the need for both subjective (self-report) and objective measurements (as used in this research) to provide new insights into the field of physical activity. 

The finding that children were able to meet recommended MVPA guidelines during school hours is encouraging, particularly as previous data [46] reported few opportunities for children to be active at school. All junior boys (100%; *n* = 5) and 88% (*n* = 4) of junior girls were found to meet the daily recommended 60 min of MVPA [1,2] within school time, but only on days that included PE. Considering that children spend only half of their waking hours within an elementary school setting [47], this one school’s contribution has been shown to demonstrate a much greater contribution than expected. The finding that on non-PE days, children were more active at a MVPA and LPA levels during recess, compared to PE days, could be due to children having different physical activity needs and habits according to the type of day. Further qualitative analysis is required to explore these behaviours and examine children’s own understanding of these differences.

For others to follow suit, the key may be for all schools to offer, and provide, as many movement opportunities as possible within and throughout the school day. This could be achieved by providing access to both small and large equipment during recess and lunch times [48] and support in how to use them, as well as enabling more movement opportunities within traditional PE lessons [49]. By introducing repeated short-term high-intensity exercises in PE, both aerobic and anaerobic fitness could also be improved [50]. Schools could potentially consider having PE lessons on more than just 2 days a week, as the data indicated that days with PE lessons particularly helped the 9–10-year-old (junior) boys reach their daily recommended MVPA levels.

Although juniors had more of an impact on their PA levels than infants, which could be due to their more advanced physical development levels and motor competencies [51], the potential for all schools to offer more movement integration [15] for their students should not be ignored. This may be integrated through the support of more time within initial teacher education [14] to consider ways in which physical activity could be used in the classroom as a teaching tool, as well as how to encourage participation in physical activity during recess and lunch times. 

This study highlights the need to educate children on the definitions and examples of differing PA intensities. Previous work has highlighted the difficulties children and adults have in understanding the differences between actual and perceived measures [3,4]. Although accelerometers are expensive and require technical expertise and additional software to analyse the data they collect effectively, they, along with other objective measures (pedometers or smart technology/smart watches), could help contribute to children’s awareness, knowledge and understanding of what they are doing [52]. As children’s physical activity tends to be spontaneous and intermittent, PA profiling is difficult [3,50,53]. Transitions between light and MVPA can be sporadic, as children move between these frequently, making it difficult to determine whether they are reaching a moderate or vigorous intensity level for sustained periods [3]. From this longitudinal data analysis, it is clear there is a need for children to be more aware of the different levels of intensity of physical activity they are undertaking. In order to facilitate this need, teachers could benefit from additional continuous professional development on physical activity to consolidate the learning of different levels of intensity [14]; to physically see examples of these within children; to share this knowledge with their students and to be supported in planning for movement integration and physical activities of different intensity throughout the school day (not just during PE).

## 5. Strengths and Limitations

This study is limited due to it only providing detailed APA and PPA of one case study school setting. However, focusing on physical activity within school hours is multidimensional and, as it was monitored objectively over a longitudinal time period, allowed for depth of data for this educational setting, isolating the variables of socio-economic background, curriculum and influence of recess and lunch times. Although this study is limited in terms of generalisability beyond the population represented, the conclusions and data provide an important insight that can be linked back to the rest of the children within the same classes, as they would all be experiencing the same curriculum, format of the day and have the same movement opportunities. The findings could inform a larger study examining more schools from different economic and locational settings. 

Self-reporting has numerous limitations in that children can find it difficult to accurately recall [3]. Yet, from an educational perspective, it is important for children to learn to articulate and understand how their bodies are responding to physical activity, so it is vital to explore their recall if teachers are to support them in their development and understanding of different physical activity intensities. Schneider et al. [54] do acknowledge that physical activity has been successfully assessed using questionnaires but acknowledge that it can be a highly complex cognitive task, which young children may struggle with.

The use of a 60 s epoch recording was chosen as this is the recommended way to assess the physical activity levels of children [33,34,35]; particularly in longitudinal data collection. However, caution is aired [55] in the use of a 60 s epoch for future research, due to the misclassification of intensity levels, particularly at vigorous levels, and the potential underestimation of time spent engaging in vigorous physical activity. 

## 6. Conclusions

This longitudinal case study has illustrated that schools can be key places for children to be physically active throughout the school day, as well as the significant impact that PE days and lessons can have on older children’s overall physical activity levels. Yet, children lack the ability to accurately perceive their APA levels. If children can begin to understand and be aware of the intensity of their PA more, this could help them to better implement its health benefits into their lives. Furthermore, if teachers were supported in learning about the different intensity levels of children’s physical activity and how to embed these into their work, they could be better able to support, emphasise and deliver PA to their students throughout the school day. 

## Figures and Tables

**Table 1:** Total number of minutes of for each part of the school day according to type of day and age group.

	Time (mins)	Time (mins)	Time (mins)	Time (mins)
Year Group	Infants	Infants	Juniors	Juniors
Part of Day/Type of Day	PE days	Non-PE days	PE days	Non-PE days
Curriculum time	236	276	251	291
Morning recess	20	20	20	20
Lunch time	60	60	60	60
Afternoon recess~	15	15	~	~
PE	40	*	40	*

PE = Physical Education. ~ indicates that afternoon recess only occurred from infants. * indicates that there are no data as PE lessons did not exist on non-PE days.

**Table 2:** Different physical activity intensity levels during PE and non-PE days by age and sex.

Participant	MVPA *		LPA **	
	PE	Non-PE	PE	Non-PE
Morning recess (minutes)				
Infants Boys	10 ± 4	8 ± 3	8 ± 3	10 ± 2
Infants Girls	8 ± 2	5 ± 1	9 ± 2	11 ± 1
Juniors Boys	10 ± 3	10 ± 4	7 ± 2	6 ± 2
Juniors Girls	8 ± 3	8 ± 4	7 ± 2	7 ± 2
Lunch time (minutes)				
Infants Boys	15 ± 6	15 ± 7	22 ± 3	23 ± 3
Infants Girls	11 ± 4	11 ± 4	21 ± 2	23 ± 3
Juniors Boys	23 ± 7	22 ± 7	19 ± 3	19 ± 4
Juniors Girls	19 ± 8	19 ± 7	21 ± 4	20 ± 4
Afternoon recess (minutes)				
Infants Boys	3 ± 1	3 ± 1	6 ± 1	6 ± 1
Infants Girls	2 ± 1	3 ± 1	6 ± 0	6 ± 1

* MVPA = moderate to vigorous physical activity levels. ** LPA = light physical activity levels. PE = Physical Education.

## Data Availability

The data presented in this study are available on request from the corresponding author.
